# Signal Reconstruction of Pulmonary Vein Recordings Using a Phenomenological Mathematical Model: Application to Pulmonary Vein Isolation Therapy

**DOI:** 10.3389/fphys.2017.00496

**Published:** 2017-07-17

**Authors:** Harry D. Green, Glyn Thomas, John R. Terry

**Affiliations:** ^1^College of Engineering, Mathematics and Physical Sciences, University of Exeter Exeter, United Kingdom; ^2^Wellcome Trust Centre for Biomedical Modelling and Analysis, University of Exeter Exeter, United Kingdom; ^3^Living Systems Institute, University of Exeter Exeter, United Kingdom; ^4^Bristol Heart Institute Bristol, United Kingdom; ^5^EPSRC Centre for Predictive Modelling in Healthcare, University of Exeter Exeter, United Kingdom

**Keywords:** atrial fibrillation, radiofrequency ablation, pulmonary vein isolation, mathematical model, pulmonary vein recording, signal reconstruction, minimal cardiac models, cardiology

## Abstract

Atrial fibrillation (AF), the most prevalent cardiac arrhythmia, is commonly initiated by ectopic beats originating from a small myocardial sleeve extending over the pulmonary veins. Pulmonary vein isolation therapy attempts to isolate the pulmonary veins from the left atrium by ablating tissue, commonly by using radiofrequency ablation. During this procedure, the cardiologist records electrical activity using a lasso catheter, and the activation pattern recorded is used as a guide toward which regions to ablate. However, poor contact between electrode and tissue can lead to important regions of electrical activity not being recorded in clinic. We reproduce these signals through the use of a phenomenological model of the cardiac action potential on a cylinder, which we fit to post-AF atrial cells, and model the bipolar electrodes of the lasso catheter by an approximation of the surface potential. The resulting activation pattern is validated by direct comparison with those of clinical recordings. A potential application of the model is to reconstruct the missing electrical activity, minimizing the impact of the information loss on the clinical procedure, and we present results to demonstrate this.

## Introduction

Cardiac disease is the most common cause of death among the adult population worldwide (Murray and Lopez, 1997). Of the main contributors to cardiac disease, atrial fibrillation (AF) is the most common arrhythmia (Kannel et al., 1998), with a lifetime incidence of one in four at age 40 (Lloyd-Jones et al., 2004) and prevalence aged 80+ of approximately 9%. AF is associated with a near doubling of mortality (Benjamin et al., 1998) due primarily to a three-fold increase in the likelihood of congestive heart failure and a five-fold increase in the likelihood of stroke (Camm et al., 2012). Consequently, AF is a significant burden on public health. For example, in the UK the cost of treating cases of AF and complications thereof are estimated at £2 billion annually (The Office of Health Economics, 2009), whilst in the USA AF is predicted to double in prevalence from 2010 to 2030 (Colilla et al., 2013). AF is characterized by a rapid, irregular, atrial rate due to spiralling wavefronts (Jalife, 2003; Nattel et al., 2008; Calvo et al., 2014), and is most commonly initiated from a small section of the left atrial myocardium that extends over the base of the pulmonary veins [responsible for an estimated 88% (Chen et al., 1999) to 94% (Haissaguerre et al., 1998) of cases].

Herein we focus on Circumferential Pulmonary Vein Isolation (CPVI), a minimally invasive surgical technique for treatment of AF, in which a circular lesion is formed surrounding the pulmonary vein via the application of radiofrequency energy, electrically isolating the left atrium from the pulmonary vein and so preventing the propagation of an action potential (AP) in or out of the myocardial sleeve. Whilst the initial success rate of pulmonary vein isolation is approximately 85% (Bänsch et al., 2013), recurrence rates 5 months after ablation therapy can be as high as 30% in paroxysmal AF patients or 78% in permanent AF patients (Oral et al., 2002). It is desirable to ensure that the ablation process is completed as quickly as possible, as the duration of the procedure is known to strongly correlate with the rate of recurrence (Shim et al., 2013). Additionally, ablation of the pulmonary veins carries a risk of pulmonary vein stenosis (Robbins et al., 1998) and if complete electrical isolation is not achieved, the lesions can become pro-arrhythmic through the creation of conduction obstacles that facilitate the initiation of re-entrant waves.

**Figure 1:**
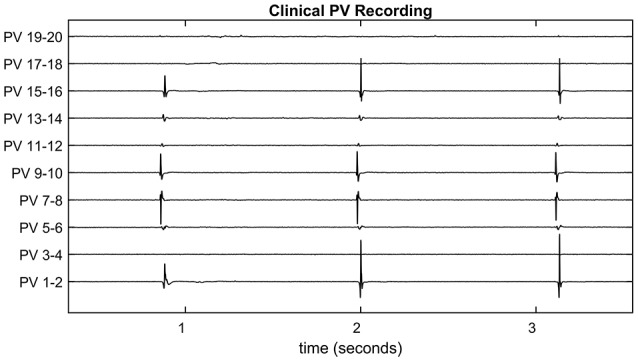
Clinical pulmonary vein recording. The pulmonary vein recording of a patient with atrial fibrillation during pulmonary vein isolation therapy. Spiking indicates electrical activity as the action potential propagates through the recording catheter. Since the pulmonary vein is not a perfect cylinder, not all electrodes make a good contact. For example, Channels 3-4, 17-18, and 19-20 show no spiking activity for this reason. These are referred to as missing channels throughout the paper.

It is common for the initial circular lesion made during CPVI to be incomplete and small conduction gaps remain. These are most commonly due to poor depth penetration of the lesion and the ablation catheter not maintaining a continuous contact with the heart tissue. To provide a guide to the surgeon as to the location of the conduction gaps, bipolar recordings of electrical activity around the pulmonary vein are taken using a lasso catheter typically consisting of 10 or 20 electrodes (see Figure 1 for an exemplar time-trace). The conduction gap is assumed to correspond to the location of the electrode(s) where the first spikes are observed and these sites are targeted for further ablation (Haissaguerre et al., 1998; Haïssaguerre et al., 2000). However, as the pulmonary vein is not a perfect cylinder it is common for some electrodes to make poor contact with the tissue. Figure 1 is an example of this happening in clinic, and in this case it is difficult to infer the activation pattern across PV 17-18 and 19-20. If these missing channels correspond to the region of first activation, this information loss could potentially lead to ablating the wrong region, or concluding the process has been successful.

In this paper, we focus on developing a mathematical representation of the phenomenology of the electrical signal recorded from the lasso catheter and to use this to reconstruct missing electrical signals. This is in contrast to typical approaches to modeling the cardiac AP or the body surface ECG where physiological detailed models are typically used (see, for example, Clayton et al., 2011; Noble et al., 2012 for comprehensive reviews). Developed appropriately, phenomenological models can be used to produce patient-specific simulations of the electrophysiology during treatment and could therefore form a part of a therapeutic decision support system to minimize the impact of information loss in clinic. This approach is motivated by our experience in neurology, where mathematical models of the phenomenology of electrical recordings from scalp electroencephalography have demonstrable potential in providing decision support for the diagnosis of epilepsy, without recourse to detailed models of the underlying neurophysiology (Schmidt et al., 2016).

The use of physiologically detailed mathematical models has enabled personalized 3D modeling of the atria, largely involving detailed biophysical models to investigate mechanisms behind the sustenance of AF (McDowell et al., 2013; Zahid et al., 2016). Additionally, fibrosis patterns have attracted significant recent attention (McDowell et al., 2012), and results obtained from the detailed models have elucidated the role of so called “islands of fibrosis” in the atria (Chrispin et al., 2016). Further, techniques are in place for the simulation of “virtual ablation” and bipolar electrograms (Dang et al., 2005; Reumann et al., 2008; Tobon et al., 2010; Yun et al., 2014). In a 2014 study (Hwang et al., 2014) a variety of ablation strategies were simulated and compared in a computational study, finding that CPVI with two additional linear lesions (along the roof and posterior wall) showed the highest AF termination rate.

However, such studies typically make the following assumptions:

1. the data collected and used to constrain the model is the ‘ground truth’;
2. ablated lesions made by the cardiologist are continuous.

Both assumptions are likely to be invalidated in the clinical setting, where significant information loss due to poorly connected electrodes is commonplace and conduction gaps create discontinuous lesions. These were highlighted in 2011 by Miyamoto et al. (2011) who proposed a method to infer a pulmonary vein activation map via gentle movement of the catheter. In conclusion they raised concerns that signals were unreliable due to some electrodes touching the endocardium whilst others did not. A further issue is that bipolar electrodes located symmetrically to a conduction gap will record a zero signal despite a wavefront passing through.

To address these challenges, we introduce a phenomenological reaction-diffusion model of the cardiac AP [the so-called Bueno-Orovio, Cherry and Fenton (BOCF) model Bueno-Orovio et al., 2008] on a cylinder with regions of zero conduction representing ablated tissue to build simulated representations of the bipolar signals recorded by the lasso catheter. Our focus on a simplified model of the phenomenology of the electrical signal, rather than a detailed model of the underlying electrophysiology, is two-fold. First, a cardiologist uses information from the macroscopic electrical recordings to identify appropriate site(s) to ablate, without recourse to any detailed understanding of the underlying electrophysiology. Second, the time available for the surgical procedure is of the order 1 h meaning that the model must efficiently reproduce a signal to be of use as a decision support tool during the procedure. The BOCF model provides a pragmatic balance between the quality of the simulated signal and the computational time required to produce the output. For example, many detailed biophysical cardiac models, such as Courtemanche et al. (1998); Nygren et al. (1998); Priebe and Beuckelmann (1998); Iyer et al. (2004); ten Tusscher (2004) require significant time (of order hours) to compute appropriate APs, rendering them inappropriate in the clinical setting. In contrast, the BOCF model can be run multiple times for parameter estimation and sensitivity analysis over much shorter timescales (of order seconds to minutes). There exist models, verified either with data or by their to the output of detailed biophysical models that satisfy these conditions (Mitchell and Schaeffer, 2003; Bueno-Orovio et al., 2008; Fenton and Cherry, 2008).

We demonstrate that this simple model can reproduce the activation pattern across electrodes recorded in clinic. Furthermore, we test the potential of the model to reconstruct recordings that have been lost to poor contact. We verify the accuracy of the simulated recording using clinical data and minimizing the root mean squared error between the activation patterns in the model and those in the data. To test the accuracy of the reconstruction, we use recordings for which all channels are spiking cleanly, and remove a subset, so that the original signal can be used for error calculation. Further, we present results showing cases in which the reconstruction of signals via the model would lead to reducing the number of RF pulses. Reducing the number of RF pulses would both minimize unnecessary damage to the heart and shorten the duration of the procedure. This is significant due to the correlation between the duration of the procedure and the rate of recurrence (Shim et al., 2013). Finally, as we are motivated by the ultimate potential for clinical applicability, we also demonstrate a small trial which shows (a) that the loss of information affects the decision of the cardiologist, and (b) that the magnitude of this effect is reduced when the reconstructed signals are provided to the cardiologist.

## Methods

**Figure 2:**
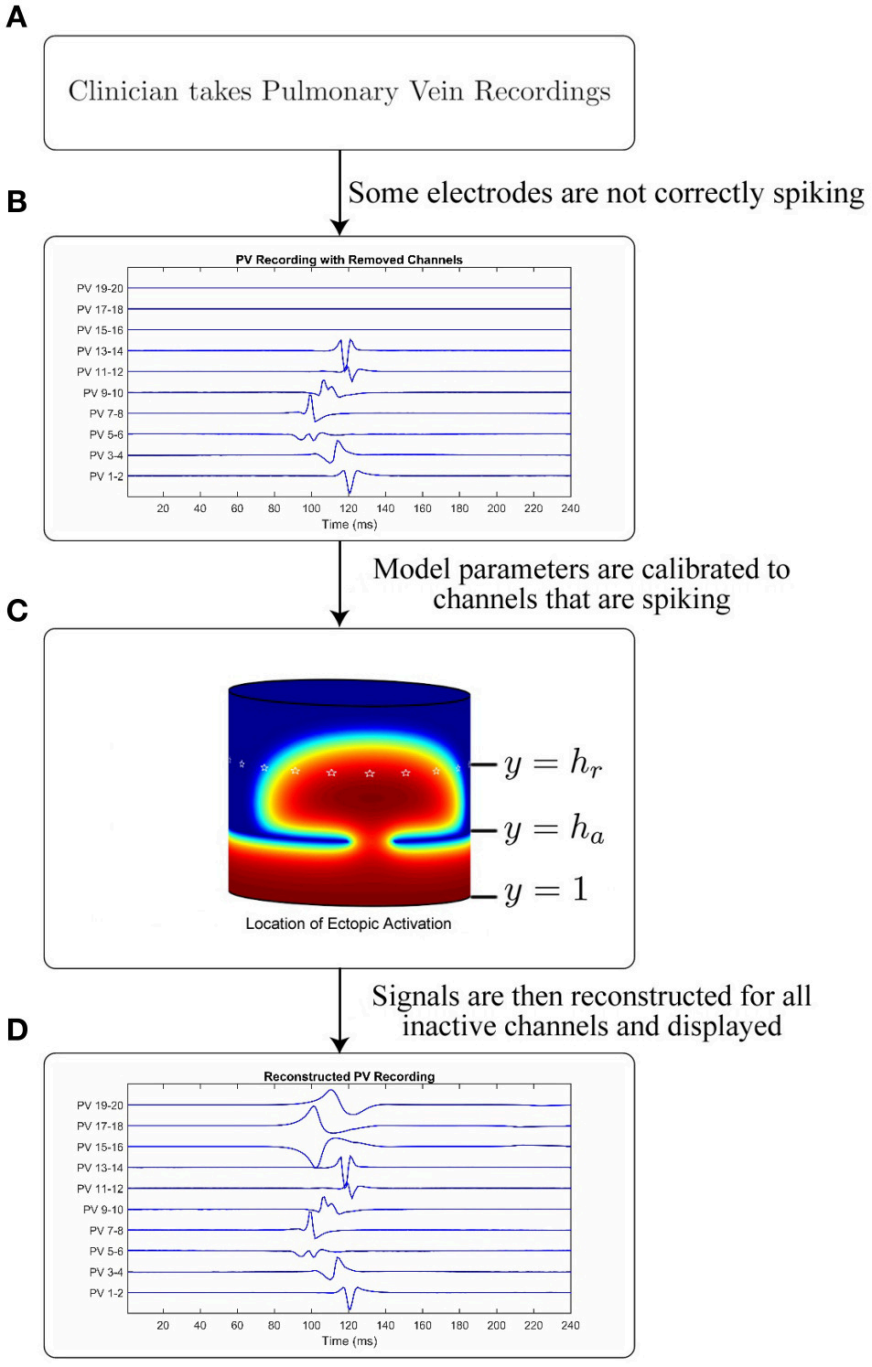
Schematic and application of model. Figure showing the intended application of the model. **(A)** A cardiologist records Pulmonary Vein Recordings using a lasso catheter. **(B)** An example of a pulmonary vein recording with some channels not correctly spiking (missing channels). **(C)** A model simulation demonstrating the propagation through a conduction gap, showing high intracellular potential in red and low in blue. White stars are plotted on the electrode locations. **(D)** The result of applying the model to the signal in B to recover the lost channels.

In this section we introduce the mathematical model used to generate the underlying AP which is in turn used to generate a traveling wave of intracellular potential within the pulmonary vein. We describe the methods used to simulate the models and how their parameters may be calibrated (either from synthetic data or clinical recordings). We further describe how the model can be used to reconstruct missing channels from data collected clinically from a lasso catheter. A schematic of how the overall process might be used to provide clinical decision support is illustrated in Figure 2.

### Mathematical model of the underlying AP

In the current paper we model the pulmonary vein AP using an extension of the 1998 Fenton-Karma model (Fenton and Cherry, 2008): the four variable Bueno-Orovio Cherry Fenton (BOCF) model. This is a monodomain phenomenological model of the human ventricular AP first introduced in Bueno-Orovio et al. (2008):

(1) $$\begin{array}{l} \;\dot{u}=\nabla \cdot (D_{BOCF}\nabla u)-(J_{fi}+J_{so}+J_{si})\\ \;\dot{v}=[1-H(u-\theta_{v})](v_{\infty }-v)/\tau_{v}^{-}-H(u-\theta_{v})v/\tau_{v}\\ \dot{w}=[1-H(u-\theta_{w})](w_{\infty }-w)/\tau_{w}^{-}-H(u-\theta_{w})w/\tau_{w}\\ \dot{s}\;=((1+\tanh[k_{s}(u-u_{s})])/2-s)/\tau_{s}. \end{array}$$

Here *u* represents the transmembrane voltage, *J*_fi_, *J*_so_ and *J*_si_ are phenomenological summations of the fast inward, slow outward, and slow inward currents respectively. *J*_fi_ is effectively gated by the gating variable *v*, *J*_so_ is voltage gated, and *J*_si_ is effectively gated by the product of the gating variables *w* and *s. D*_*BOCF*_ is either a spatially dependant diffusion constant (under the assumption of isotropic diffusion), or a diffusion tensor (under the assumption of anisotropic diffusion). We always take initial conditions at the resting state, where [*u*(0), *v*(0), *w*(0), *s*(0)] = [0, 1, 1, 0]. A full description of this model can be found in Bueno-Orovio et al. (2008).

### Calibrating BOCF model parameters

**Figure 3:**
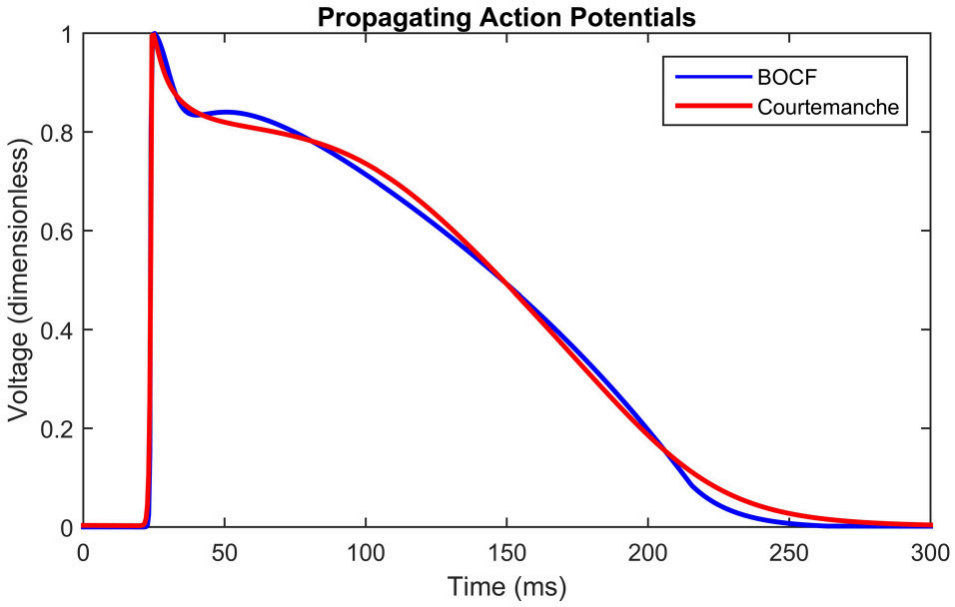
Propagating action potentials. Plots of Courtemanche (Courtemanche et al., 1998) (blue), and BOCF (Bueno-Orovio et al., 2008) (red) models using the parameters in Table 1, of an AP at a point 10 mm from the stimulus with a spatial resolution of 0.2 mm and a time step of 0.01 ms. Model calibrated by minimizing the root mean squared error using the Nelder-Mead method. The Courtemanche model is solved using the parameters in Courtemanche et al. (1999).

Given that the shape of the emergent electrical activity recorded on the lasso catheter may be constrained by the underlying structure and function of the AP, a propagating AP was simulated using the detailed biophysical Courtemanche model for the human atrium (Courtemanche et al., 1998; Imaniastuti et al., 2014; Labarthe et al., 2014) as a proxy for clinical AP data. A generic AP from the Courtemanche model was modified to account for the electrical remodeling associated with AF (Courtemanche et al., 1999) and used as the initial stimulus for the BOCF model with parameters as defined in the sample fitting code in the appendix of Bueno-Orovio et al. (2008). These parameter choices were then evolved using the Nelder-Mead Simplex Algorithm (Nelder and Mead, 1965) (implemented by MATLAB's fminsearch), by minimizing the root mean squared error between subsequent APs (see Figure 3). With a spatial resolution Δ*x* = 0.2 mm, a diffusion constant of *D*_*Court*_ = 2.615 was necessary for the simulated wavefront to match the conduction velocity of 48 cm/s observed clinically (Labarthe, 2013). To eliminate any effects from boundary conditions or transients from the stimulus, the fit was performed at the point *x* = 10 mm on a tissue cable 20 mm long. A cycle length of 600 ms was used to match the clinical data. This process resulted in the parameter choices defined in Table 1.

**Table 1:** Parameter values of Bueno-Orovio Cherry Fenton model.

**Parameter**	**BOCF**	**BOCF-AF**	**Parameter**	**BOCF**	**BOCF-AF**
$\tau_{v}^{+}$	1.6650	1.6234	τ_*so*2_	1.0261	0.9862
$\tau_{w1}^{-}$	82.6769	69.1816	*k*_*so*_	2.0487	2.3769
$\tau_{w2}^{-}$	9.0959	14.1985	*u*_*so*_	0.5149	0.9220
$k_{w}^{-}$	63.8099	65.4466	τ_*s*1_	2.5879	2.5603
$u_{w}^{-}$	0.0331	0.0316	τ_*s*2_	18.5596	12.5106
$\tau_{w}^{+}$	213.1962	140.2385	*k*_*s*_	2.0468	1.5749
τ_*fi*_	0.1256	0.0990	*u*_*s*_	0.7033	1.1640
τ_*o*1_	431.0734	452.4879	τ_*si*_	2.1260	2.1756
τ_*o*2_	6.5724	5.5292	τ_*w∞*_	0.0637	0.0601
τ_*so*1_	33.2039	25.6007	$w_{\infty }^{⋆}$	0.6520	0.9408
*D*_*BOCF*_	N/A	0.8314			

*The original parameters of the Bueno-Orovio Cherry Fenton model (Bueno-Orovio et al., 2008) alongside the parameters obtained from our fitting algorithm. Diffusion coefficient D_BOCF_ was not given in the original model*.

Figure 3 shows the shape of the propagating APs under the above conditions using the BOCF model with parameters as in Table 1, alongside the Courtemanche AF model as described in Courtemanche et al. (1999). The important qualities reproduced were conduction velocity (indicated by the simultaneous spike), upstroke velocity, and AP duration.

For the case of anisotropic diffusion an asymmetric finite difference method was used to simulate the BOCF model (see van Es et al., 2014 for full details). Since in general the degree of anisotropy for an individual patient is unknown, we included the principal axes and eigenvalues of the diffusion tensor as additional parameters to be optimized by our fitting algorithm. Physiological studies place the anisotropy ratio between 2 and 10, (Koura et al., 2002; Xie and Zemlin, 2016), which were used as bounds in our algorithms. The initial principal axes were placed at 45 degrees to the *x* and *y* axes, maximizing the effect on the propagation pattern.

### Simulating pulmonary vein recordings

2D simulations of the pulmonary vein were performed by numerical integration of Equation (1) by a finite difference method over a discretized cylindrical domain to represent the excitable myocardial sleeve extending over the base of the pulmonary vein. Dimensions vary from vein to vein, with the right inferior typically the largest and the left inferior the smallest (Stojanovska and Cronin, 2008). We assume dimensions within the range of observed measurements: a length of 15 mm (Cronin et al., 2007) and a diameter of 12.5 mm (Cabrera et al., 2002; Kim et al., 2005). A spatial resolution of Δ*x* = 0.2 mm was used to discretize this cylinder into a rectangular domain of 200 × 75 grid points. Periodic boundary conditions were used along the lines *x* = 1 and *x* = 200, whilst Neumann boundary conditions were used along the lines *y* = 1 and *y* = 75 (where *x* and *y* represent nodes on the grid). We set the conductivity to 0 to model the effect of lesions due to ablated tissue at the relevant points, following the approach introduced in Dang et al. (2005); Reumann et al. (2008); Tobon et al. (2010). As we are only concerned with the effect on the AP propagation from the ablation process, we do not require a model of the thermodynamic processes of the catheter itself (Berjano, 2006; Suárez et al., 2010).

A visual representation of this structure is shown in Figure 2, which shows the propagating intracellular potential with the lines *y* = 1, *y* = *h*_a_, and *y* = *h*_r_ annotated (Figure 2C). An ectopic is initiated from a stimulus along the line *y* = 1; the edge of the myocardial sleeve furthest from the atrial junction. Virtual ablation is performed by introducing a line of lesions on the circle *y* = *h*_a_ such that *D*(*x, h*_a_) = 0. Conduction gaps are modeled such that *D*(*x, h*_a_) = *D*_*BOCF*_ (for conductive tissue on small segments of the circle *y* = *h*_a_). Consequently, semi-circular wavefront(s) will form on the other side of the lesions. Although loosely based on the underlying mechanisms, the values of the obtained parameters are phenomenological, and fit to the available data to ensure an accurate simulation on the lasso catheter electrodes, not to provide an estimation of the real location of the conduction gap.

We simulate pulmonary vein recordings from the lasso catheter across *n* electrodes (where *n* is typically 10 or 20), on *y* = *h*_r_, where *h*_r_ > *h*_a_. The electrodes are assumed to be equally spaced *d* = 200/*n* apart, such that for an *n* electrode catheter *c* = (*a, h*_r_) where *a* = {*d*, 2*d*, …, *nd*}). At each point *c* = (*x*′, *y*′), an approximation for the surface potential Φ described originally in Gima and Rudy (2002) is given by:

(2) $$\begin{array}{r} \Phi \left(x^{\prime },y^{\prime }\right)=aD\left(x^{\prime },y^{\prime }\right)\int \int \left(-\nabla u\right)\cdot \left[\nabla \frac{1}{r}\right]dxdy, \end{array}$$

where

(3) $$\begin{array}{r} r=\sqrt{{\left(x^{\prime }-x\right)}^{2}+{\left(y^{\prime }-y\right)}^{2}}. \end{array}$$

Bipolar recordings between electrodes *i* and *j* (denoted PV i-j clinically) are simulated by:

(4) $$\begin{array}{r} PVi-j=\Phi \left(a_{i},h_{r}\right)-\Phi \left(a_{j},h_{r}\right). \end{array}$$

Throughout this paper, we divide the pulmonary vein into three equal sections, with the ablation line positioned at *h*_a_ = 25 and the recording catheter positioned at *h*_r_ = 50. This is a practical consideration, as quantifying these measurements during the procedure would be difficult given information collected as standard in clinical practice.

### Relative activation time curves

The important characteristics of both the simulated and recorded data are the activation times (from maximal absolute value of *dV*/*dt*) of each signal compared to the others, as this gives a representation of the wavefront shape termed the *relative activation time curve*. It is necessary to use the absolute value as the recordings are bipolar. The relative activation time curve can be visualized by plotting the catheter along the *x* axis and its activation time on the *y* axis, giving a curve of the activation times of each signal relative to the others.

To understand the relationship between the relative activation time curve and parameters of the overall pulmonary vein model, the quantity, size and locations of conduction gaps are used as input parameters, since these have the most profound effect on the emerging wavefront shape. The root mean squared error between relative activation times obtained from simulated and clinical recordings are minimized, again using the Nelder-Mead Simplex Search method (implemented by MATLAB's fminsearch) to establish the location of conduction gaps which result in the most accurate activation time curve. Here it is important to note we do not claim to have found the location of the conduction gap(s) via this fit, only that we have calibrated model parameters that most closely recreate the phenomenology of the waveforms from the recording catheter.

### Reconstruction of missing electrodes

In the cases for which there is poor contact between recording catheter and tissue, the signal is typically flat or white noise. This is evident, for example, in channels PV 3-4 17-18, and 19-20 in Figure 1. To reconstruct missing electrode recordings, a partial relative activation time curve was obtained from the active channels. Model parameters of the overall pulmonary vein model were calibrated from the active channels, using the Nelder-Mead Simplex Search (implemented by MATLAB's fminsearch).

### Clinical data

Pulmonary vein recordings used in this paper were obtained from adult male and female subjects undergoing pulmonary vein isolation therapy at Bristol Heart Institute. Bipolar recordings were obtained from a deflectable, circular, 20-pole Lasso catheter (Biosense Webster Ltd). Patients with both paroxysmal and persistent AF were included but all cases were paced into normal sinus rhythm by pacing at 600 ms intervals, as per standard clinical practice. All data were appropriately anonymized prior to their use in this study. Under United Kingdom law, patient data collected during normal clinical routine and anonymized before research use may be used for research without additional consent.

## Results

### Simulated pulmonary vein recordings

First we consider how well the model can reproduce the phenomenology of the pulmonary vein recordings when all 10 channels are active. To consider this, we use an exemplar set of clinical pulmonary vein recordings collected during pulmonary vein isolation therapy (as described in the methods). The goodness of fit between clinical recordings and model simulations is determined by minimizing the root mean squared error between the relative activation times of the model and the data. This is achieved by varying the positions of conduction gaps in the model. The average of ten recorded events in the data is used to form the target relative activation time curve. This ensures some robustness to variation in the data and enables us to estimate the conduction gap location and width as parameters, which should be constant until ablated.

**Figure 4:**
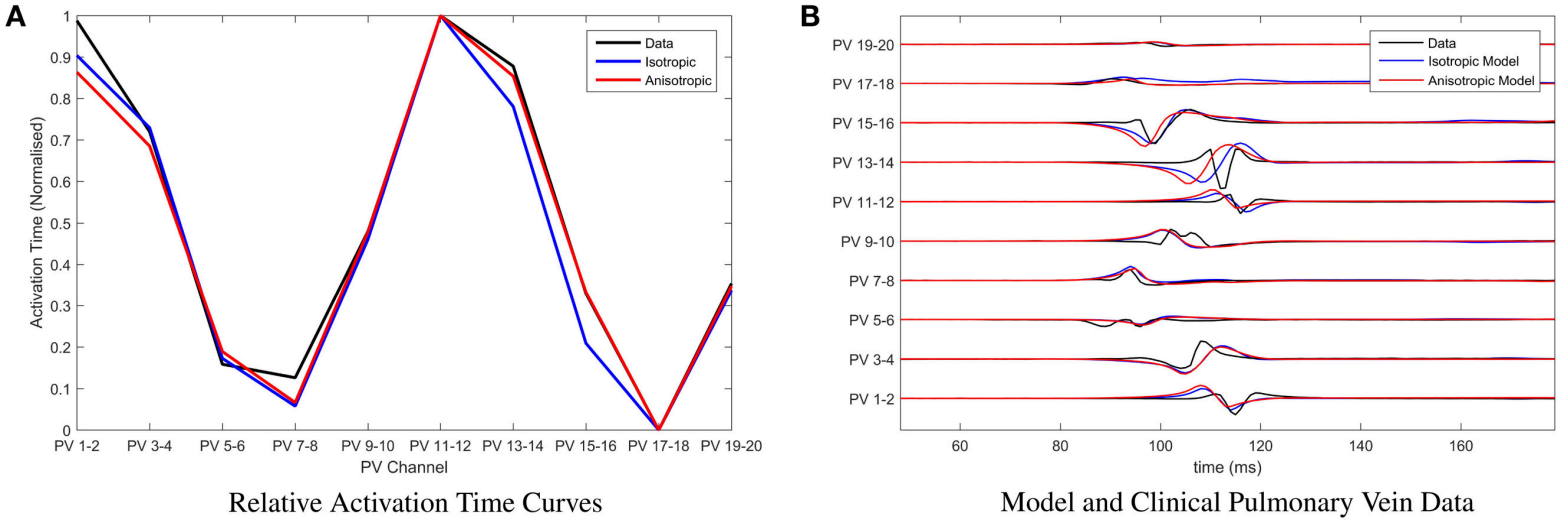
Parameter fitting and comparison with clinical recordings. **(A)** Presenting the relative activation time curves for the clinical data (presented in full in **B**) (black), the model assuming isotropic diffusion (blue) and the model assuming anisotropic diffusion (red). Parameters of the model were calibrated by minimizing the root mean squared error between clinical recording and the model using the Nelder-Mead method. **(B)** Presenting an exemplar of clinical pulmonary vein recordings collected during pulmonary vein isolation therapy at the Bristol Heart Institute (black). Overlaid are the model simulations, with parameters calibrated as described in **(A)**, under the assumption of isotropic diffusion (blue) and isotropic diffusion (red) respectively.

For the chosen clinical data, and for parameter choices of the underlying BOCF model as in Table 1, we find that the root mean squared error between the relative activation time curve of the clinical data and that of the model (assuming isotropic diffusion) is minimized by placing conduction gaps centered on points *x* = 65.5 and *x* = 167, with widths 11 and 6 respectively. Both the number of minima and their locations are used to optimize position and width of the conduction gaps. This is important since both the number of minima and their locations within the relative activation time curve emerge as a result of the conduction gaps generating the signal. In current clinical practice, the earliest activation time(s) (e.g., the minima of the relative activation curve), are the most important, as these are assumed to be closest to the conduction gap and therefore the optimal ablation site. This is illustrated in Figure 4A, where we also present a model fit under the assumption of anisotropic diffusion. In this case the conduction gaps are centered on points *x* = 59.5 and *x* = 164.5, with width 11 and 13 respectively.

In Figure 4B, we present a comparison between the original choice of clinical pulmonary vein recordings and simulations for the two classes of model. Time units of the model are rescaled such that the relative activation time-scale of the model is equivalent to that of the clinical recordings, which permits a clearer visual comparison. Note that both classes of model result in visually similar simulated pulmonary vein recordings. We perform a more rigorous analysis of differences between anisotropic and isotropic diffusion later, when considering the ability of the model to reconstruct missing channels in the clinical data.

**Figure 5:**
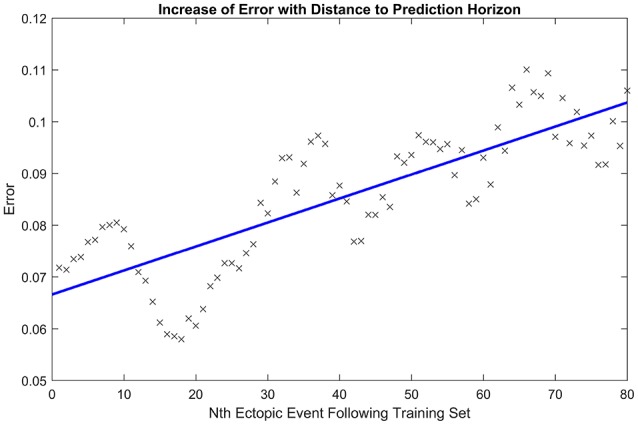
Increase of error with prediction horizon. Illustrating how the root mean squared error in the activation time curve scales as the number of the ectopic event *N* increases away from the initial ten ectopic events used to calibrate model parameters. The line of best fit is displayed in blue. The periodicity in the error is likely due to rhythmic movements of the patient, such as breathing.

Next, we tested the capacity of the model to predict future ectopic events, given an average over an initial ten events. For the identified choice of model parameters from the initial ten events, we simulated a series of additional ectopic events and for each event we calculated the root mean squared error between relative activation time curves obtained from either simulated or clinical ectopic events. We define *t*_0_ as the time of the last event in the training set, and *t*_*N*_ as the time of the *N*^th^ subsequent ectopic event. Figure 5 shows how this error scales as the number *N* of the ectopic event moves further away from the training set. The apparent periodicity in the error is most likely due to rhythmic movements of the patient, such as breathing.

### Reconstructing missing channels

We now focus on the capacity of the model to reconstruct missing channels, exemplars of which were shown in Figure 1. This is a key result of this paper, and the one with most relevance to a potential clinical decision support system. To test the accuracy of the model, we start with a clinical recording for which all channels are active. We then eliminated a subset *n* (*n* = 0 to 5) of the channels replacing them with a 0 time trace. Five was chosen as the upper limit, since clinically a recording with less than half the channels active would not be relied on for determining the site of ablation. We then estimated model parameters using the same approach as in the previous section, but only data from those channels that were active. Using these parameters we then contrasted the error between the relative activation time curves obtained from the simulated next ectopic event and the subsequent ectopic event from the original clinical recording (including all channels). This enables us to assess how well the model can reproduce clinically relevant information (since the relative activation time curve is used for determining the site of ablation).

**Figure 6:**
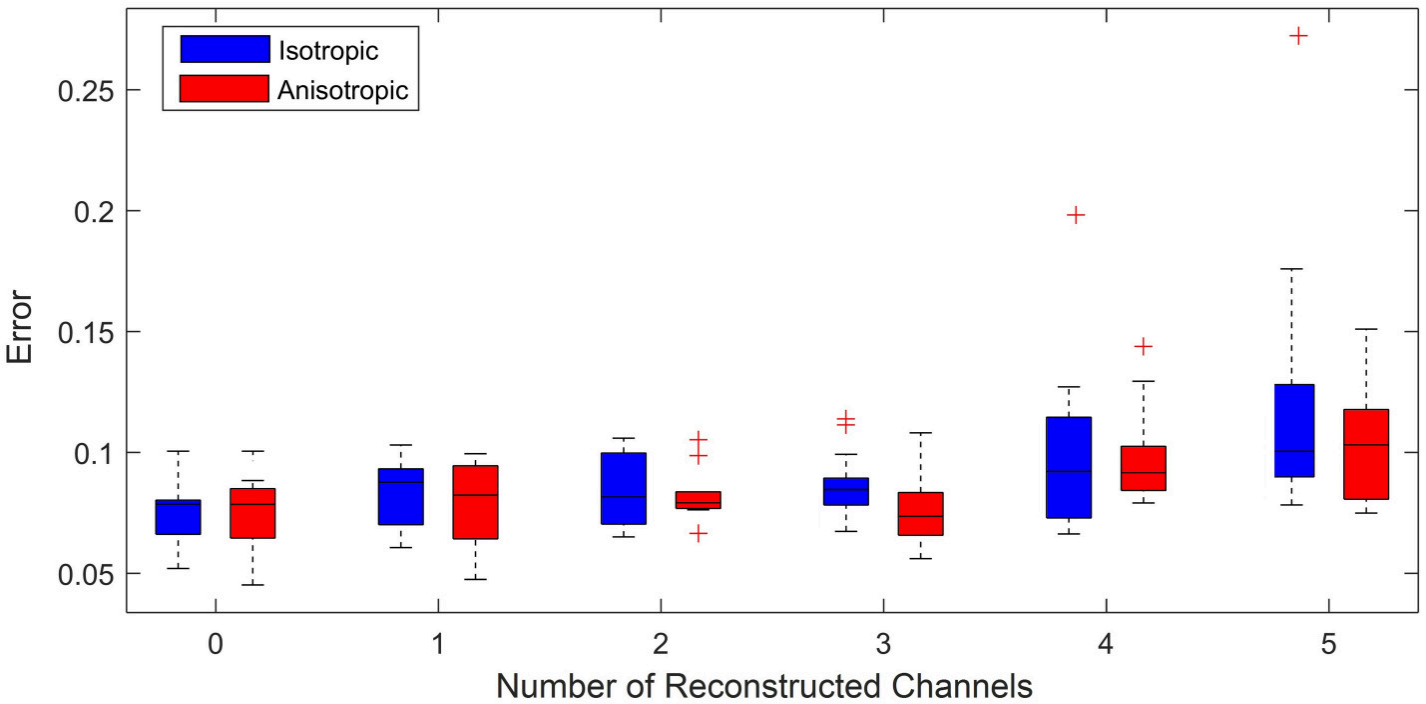
Error vs. number of reconstructed channels. Box plot illustrating how increasing the number of missing channels influences the minimized root mean squared error between the relative activation time curves obtained from the model (assuming both anisotropic diffusion (red) and isotropic diffusion (blue)) and the original clinical recording. For each case 20 simulations for up to 20 random choices of channels to be reconstructed were performed. (+) symbols denote outliers in the 1% tail of the error distributions. The case of 0 reconstructed channels enables a comparison of the limit of goodness of fit between models and the clinical data. We see that for 3 or fewer channels being reconstructed, uncertainty is predominantly due to model choice rather than the number of reconstructed channels, as we see no significant change in the mean error between model and clinical data. We use an unequal variances *t*-test to determine whether the errors came from a distribution with equal mean and find that with the exception of 3 channels reconstructed there is no significant difference in the errors between models.

Figure 6 shows a box plot for each value of *n*. Each box in the box plot represents the root mean squared error between the relative activation time curves obtained from the average across 20 model simulations (with anisotropic diffusion and without) and that obtained from a clinically recorded ectopic event. The case *n* = 0 enables us to consider the limit of the goodness of fit between the model and the clinical data. This is effectively the intrinsic error attributable to the choice of model. For subsequent plots, *n* random channels were removed from the training set (simulating the effect of lost information due to poor contact). Different time intervals and different signals were used for each calculation so that the error distribution presented is as close as possible to the errors that we might expect to observe in clinic. This is important as it minimizes the likelihood of observations simply being due to an artifact of the ectopic event chosen for the fit.

For up to 3 channels reconstructed, the median and maximum errors do not significantly increase over that of the control whether or not anisotropy is considered. This is an important result as it demonstrates reconstructing up to three missing channels is not a significant source of additional error and therefore the model as presented may ultimately have clinical use under these conditions. Removing more than 3 channels leads to information loss resulting in outliers with statistically significant errors (see the cases for 4 and 5 channels removed). This demonstrates the limit of the number of missing channels that the considered models can reliably reconstruct.

To consider the whether the assumption of anisotropic diffusion is significant, we performed an unequal variances *t*-test (so-called Welch's *t*-test) to test whether the errors from each model could have come from a distribution with the same mean. This test consistently showed no significant difference (*p* > 0.05), except for the case of 3 signals reconstructed (*p* = 0.0414). This suggests that whilst anisotropy is clearly important in terms of the underlying physiology, it does not significantly affect the quality of model fit to the phenomenology of the recorded signals. This is an important consideration as calibration of model parameters is more efficient under the assumption of isotropic diffusion.

### Potential clinical application

To test the potential of this technique to aid the clinical procedure, we presented a cardiologist specializing in pulmonary vein isolation therapy, with three variations of clinical recordings collected from three patients:

1. the original clinical recordings with all channels active;
2. the original clinical recordings with key channels identifying the earliest activation hidden;
3. a hybrid whereby we reconstruct channels (removed in scenario 2) using the mathematical model and present these alongside the remaining active channels.

**Figure 7:**
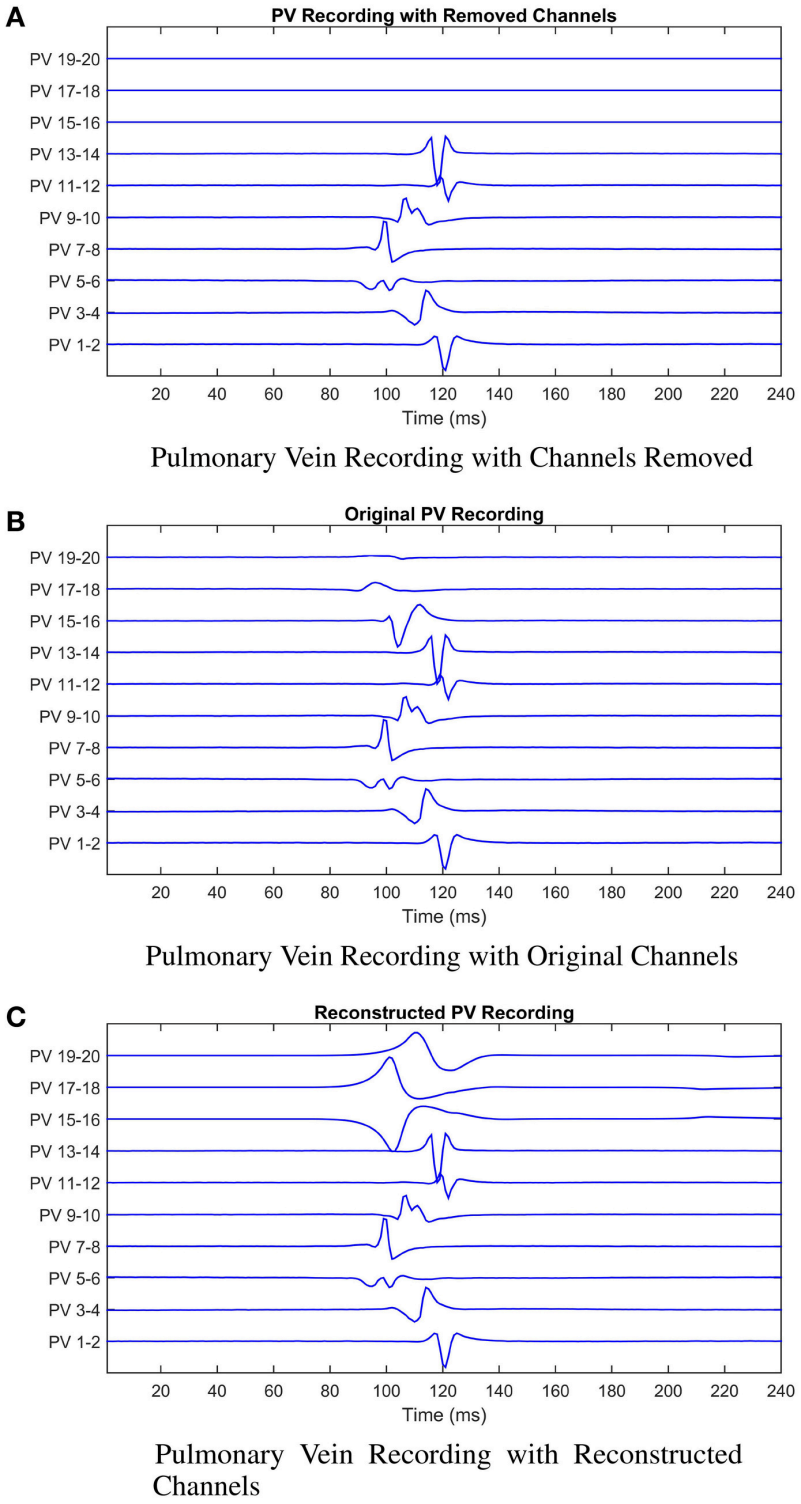
Illustrating the model reconstruction of pulmonary vein recordings. In **(A)** we present the original recording with three removed channels. In **(B)** the original recordings, and in **(C)** the original recordings combined with the reconstructed channels using the mathematical model. We presented these data to the cardiologist who made a decision about the tissue underlying the channels they would ablate in each case. This process was repeated for patients 2 and 3. In all three cases reconstruction of the missing signals resulted in an improved clinical decision in contrast to only using the recordings with missing signals.

These scenarios are illustrated in Figure 7. The cardiologist was unaware of the origin of each recording, and to avoid bias, the recordings were supplied in a random order. The following results were obtained (summarized in Table 2 for convenience). For patient 1, given the original data, the first point of ablation would have been around PV 5-6, with PV 15-16 noted as a second choice. With channels 15-16, 17-18, and 19-20 removed, only PV 5-6 was identified as the only appropriate ablation zone. When these channels were reconstructed by the model, PV 17-18 was identified as the second choice of ablation target. For this patient, the model has helped to identify a second relevant ablation target that was not identified when channels were missing. If initial ablation is not successful, the cardiologist will ablate in the area surrounding the target area, hence an initial estimate closer to the optimal location will result in successful isolation using fewer radiofrequency pulses. This will result in a smaller region of tissue being ablated and a shorter procedure.

**Table 2:** Effect of signal reconstruction on clinical decision.

**Patient**	**Missing**	**Reconstructed**	**Original**
1	PV 5-6	PV 5-6 / 17-18	PV 5-6 / 15-16
2	PV 9-10	PV 5-6	PV 5-6
3	PV 3-4	PV 9-10	PV 7-8

*The channel corresponding to the area the cardiologist would ablate given the recording with channels missing, the original data, and the data with the missing channels reconstructed via the model. The decision made with the reconstructed signals is closer to the original than the decision made using the missing recordings, demonstrating that the model reconstruction has minimized the impact of the information loss on the clinical outcome. Recordings from Patient 1 are given in Figure 7*.

In the second patient presented, a clear earliest spike time was present on PV 5-6. The removal of PV 5-6 and its neighbors led to PV 9-10 being identified as an ablation target. In this case, the reconstruction led to the same zone being targeted as the original signal, while the estimation with the recordings missing was two channels away. For this patient, reconstruction of the missing recordings led the cardiologist directly to the optimal decision.

In the final patient, the earliest spike, on PV 7-8, was removed, along with PV 5-6 and 9-10. As previously, these missing channels shifted the chosen ablation target by 2 channels. The reconstructed signal led to a target selected which was closer to the target chosen with all information present. As with patient 1, we infer this result as satisfactory, as starting closer to the optimal target will lead to quicker isolation of the pulmonary vein.

In all three cases, the missing channels influenced the decision made by the cardiologist, demonstrating the potential impact of information loss in clinic. However, when the cardiologist used the recordings combined with the signals reconstructed by the mathematical model to make a decision, the decision made was closer to the decision that would have been made had all information been present. Whilst these results provide only limited proof of concept at this stage, assuming the original data and clinical decision to represent the “ground truth,” then we believe there is significant potential for our approach to minimize the effect of this lost information.

## Discussion

In this study we have demonstrated that key features of pulmonary vein recordings can be generated by a phenomenological model, in this case the BOCF model. Calibrating parameters of the BOCF model using the post-fibrillation AP of atrial myocytes, simulated using the biophysical Courtemanche model, provides a method for rapid simulation of atrial cells afflicted by AF-induced electrical remodeling. This is in contrast to more detailed biophysical models (Courtemanche et al., 1998; Nygren et al., 1998; Priebe and Beuckelmann, 1998; Iyer et al., 2004; ten Tusscher, 2004) which may take several hours to produce an output. Given that pulmonary vein isolation therapy typically lasts at most 2 h, having a mathematical model that can run in close to real time, is a critical advantage when assessing suitability as a potential clinical decision support system.

Toward this aim, a primary result of this study was to model the phenomenology of recordings from the lasso catheter used during the pulmonary vein isolation therapy of AF. We found that the resulting model simulations accurately reproduce the relative activation time curve seen in recordings from patients undergoing this procedure. The pulmonary vein recordings made in this process are not always complete; there is often the complete loss of some of the recording channels. This is most commonly due to poor contact made between electrodes on the catheter and the pulmonary vein itself. This loss of information can result in non-optimal clinical decision making during the isolation therapy procedure. To address this issue we have demonstrated that a mathematical model fitted to the available channels of the data can be used to reconstruct those missing channels and we presented evidence in support of the accuracy of these reconstructions through comparison to clinical data. Of note, we find that up to three channels can be reconstructed without significantly increases the inherent error due to the use of a model. The results show that, in principle, these ideas could be adapted as part of a clinical decision support system, which could be run in the operating theater and provide information to the cardiologist during the procedure.

A potential limitation of this study is the loss of biological detail arising from our use of a phenomenological model over a biophysical one. However, it is important to note that the appropriateness of any mathematical model is dependent on the challenge it is designed to address. Here, we focus on the case of pulmonary vein isolation therapy, where a cardiologist is using recordings of the emergent electrical signal from the heart to make rapid decisions about regions of the heart to ablate. Consequently a model that can capture the phenomenology of these recordings (which ultimately are what the cardiologist is using to guide their decision making) is a valid approach and does not require a detailed analysis of the contribution of ionic channels and other physical quantities involved in AP propagation.

In our current model a number of assumptions have been made, most importantly regarding the conductance and the geometry. Whilst we account for anisotropic diffusion by considering additional parameters, the diffusion tensor used is still homogeneous across conductive tissue, and the wave approaching the conduction gap is planar. This is primarily since detailed fiber direction information would not be accessible to the cardiologist during the clinical procedure. A further key assumption is the approximation of the pulmonary vein sleeve as a cylinder. In the clinical procedure, the relevant region of cardiac tissue is not only the pulmonary vein sleeve, but also the atrial tissue surrounding the ostium. However, while tissue expansion and asymmetry of an anatomically accurate domain may affect the results for a given set of parameters, the signal reconstruction technique incorporates the fitting of the parameters to the available signals, which will account for the impact of these assumptions. Further geometrical assumptions, such as the angles between the incoming wave, the ablation line and the recording catheter, can not be quantified using standard clinical equipment and so we do not consider them in the current study. We also assume that all cells are free atrial wall myocytes, rather than pulmonary vein myocytes which have a shorter AP duration and amplitude in addition to a lower upstroke velocity in comparison to the left atrium (Mahida et al., 2015). However, under current clinical practice, it is not possible to identify which areas of the pulmonary vein ostium is populated by pulmonary vein myocytes as opposed to those of the atrial wall.

While the model developed in this paper has been developed with clinical applicability in mind, future work will be necessary to establish the ultimate validity of and optimize this approach in a clinical context. In particular it is important to establish the optimal level of detail of model required to reconstruct missing signals, and whether additional detail can improve the accuracy of the methods, given the constraints of time and recording protocols in standard clinical practice. Further, clinical metadata regarding the locations and times at which ablation was performed on the patient is typically not collected during the ablation procedure, so it is difficult to infer the optimal ablation zone from patient data. The availability of such data would open up many new lines of research, including the use of either phenomenological or biophysically detailed patient-specific models to estimate the optimal ablation site directly.

## Author contributions

HG and JT provided concept; designed and executed the study; analyzed and interpreted the data. GT provided data; performed analysis of clinical and reconstructed signals. All authors wrote and approved the final manuscript.

### Conflict of interest statement

The authors declare that the research was conducted in the absence of any commercial or financial relationships that could be construed as a potential conflict of interest. The reviewer NT and handling Editor declared their shared affiliation, and the handling Editor states that the process met the standards of a fair and objective review.
